# Long-term follow-up of autoimmune polyendocrine syndrome type 1 in Norway

**DOI:** 10.1210/clinem/dgag060

**Published:** 2026-02-12

**Authors:** Isil Kucuka, Anette S B Wolff, Lars Breivik, Thea Sjøgren, Eirik Bratland, Marianne Grytaas, Kari Lima, Anders P Jørgensen, Ingrid Nermoen, Åsne Bakke, Hallvard Singsås, Margrethe Svendsen, Trine E Finnes, Bjørn Gunnar Nedrebø, Thor Haug, Guri Grimnes, Marthe Landsverk Rensvik, Cecilie G Gjerde, Mihaela C Marthinussen, Marianne Øksnes, Bergithe E Oftedal, Eystein S Husebye

**Affiliations:** Department of Clinical Science, University of Bergen, 5021 Bergen, Norway; Department of Medicine, Haukeland University Hospital, 5021 Bergen, Norway; Department of Biomedicine, University of Bergen, 5021 Bergen, Norway; Department of Clinical Science, University of Bergen, 5021 Bergen, Norway; Department of Medicine, Haukeland University Hospital, 5021 Bergen, Norway; Department of Medicine, Haukeland University Hospital, 5021 Bergen, Norway; Department of Clinical Science, University of Bergen, 5021 Bergen, Norway; Department of Medical Genetics, Haukeland University Hospital, 5021 Bergen, Norway; Department of Medicine, Haukeland University Hospital, 5021 Bergen, Norway; Department of Endocrinology, Akershus University Hospital, 1474 Lørenskog, Norway; Department of Endocrinology, Oslo University Hospital, 0424 Oslo, Norway; Institute of Clinical Medicine, University of Oslo, 0424 Oslo, Norway; Department of Endocrinology, Akershus University Hospital, 1474 Lørenskog, Norway; Institute of Clinical Medicine, University of Oslo, 0424 Oslo, Norway; Department of Endocrinology, Stavanger University Hospital, 4011 Stavanger, Norway; Department of Endocrinology, St.Olavs Hospital, Trondheim 7006, Norway; Department of Medicine, Østfold Hospital, Grålum 1714, Norway; Department of Endocrinology, Oslo University Hospital, 0424 Oslo, Norway; Department of Endocrinology, Innlandet Hospital Trust, Hamar 2318, Norway; Department of Clinical Science, University of Bergen, 5021 Bergen, Norway; Department of Medicine, Haugesund Hospital, 5528 Haugesund, Norway; Department of Endocrinology, Molde Hospital, 6412 Molde, Norway; Division of Medicine, University Hospital of North Norway, 9019 Tromsø, Norway; Tromsø Endocrine Research Group, Institute of Clinical Medicine, The Arctic University of Norway, 9019 Tromsø, Norway; Department of Medicine, Ålesund Hospital, Ålesund 6017, Norway; Department of Clinical Dentistry, Faculty of Medicine, University of Bergen, 5021 Bergen, Norway; Department of Clinical Dentistry, Faculty of Medicine, University of Bergen, 5021 Bergen, Norway; Oral Health Centre of Expertise in Western Norway/Vestland, 5021 Bergen, Norway; Department of Clinical Science, University of Bergen, 5021 Bergen, Norway; Department of Medicine, Haukeland University Hospital, 5021 Bergen, Norway; Department of Clinical Science, University of Bergen, 5021 Bergen, Norway; Department of Medicine, Haukeland University Hospital, 5021 Bergen, Norway; Department of Clinical Science, University of Bergen, 5021 Bergen, Norway; Department of Medicine, Haukeland University Hospital, 5021 Bergen, Norway

**Keywords:** autoimmune polyendocrine syndrome type 1, AIRE mutations, interferon autoantibodies, cytokine autoantibodies, monogenic diseases

## Abstract

**Context:**

Autoimmune polyendocrine syndrome type 1 (APS-1) is a rare yet severe multiorgan autoimmune disease caused by mutations in the autoimmune regulator (*AIRE*) gene. Classical APS-1 arises from biallelic recessive *AIRE* mutations, whereas dominant negative mutations cause a milder, nonclassical phenotype with variable clinical presentation. Due to its rarity, long-term, population-based data are limited, underscoring the need for extended follow-up to guide lifelong care and research.

**Objective:**

To characterize the clinical profiles of APS-1 and explore associations between disease manifestations, autoantibody profiles, and AIRE mutations over an extended follow-up (1996-2025).

**Patients:**

All known Norwegian patients with APS-1.

**Methods:**

We analyzed longitudinal clinical and laboratory data of 71 APS-1 patients (49 classical, 22 nonclassical) from the Norwegian Registry of Organ-specific Autoimmune Diseases. Data included clinical progression, autoantibody and cytokine profiles, and *AIRE* genotypes. Additionally, we compared age at diagnosis of primary adrenal insufficiency (PAI) in patients with and without (n = 999) APS-1.

**Results:**

In classical APS-1, the most frequent clinical manifestations were chronic mucocutaneous candidiasis, enamel hypoplasia, and PAI, while for nonclassical APS-1 vitiligo, hypothyroidism, and PAI were most common. A broad proinflammatory cytokine signature was observed in classical APS-1, along with increased levels of the soluble form of the interferon (IFN)-α/β receptor.

**Conclusion:**

APS-1 should be considered in patients diagnosed with PAI before age 20, and *AIRE* sequencing is recommended for diagnostic confirmation. The presence of IFN-ω autoantibodies, a proinflammatory cytokine profile, and increased soluble IFN receptor levels further support the role of dysregulated interferon responses in APS-1 pathogenesis.

The autoimmune regulator (*AIRE*) plays a pivotal role in establishing central tolerance in the thymus by eliminating self-reactive T cells. Impaired AIRE function disrupts this process, allowing self-reactive T cells to escape to the periphery and reduce the pool of regulatory T cells (1). In humans, AIRE deficiency results in autoimmune polyendocrine syndrome type 1 (APS-1), either a classical form, caused by biallelic *AIRE* mutations, or a nonclassical form caused by a single dominant negative *AIRE* mutation. Both forms are characterized by organ-specific autoimmunity affecting multiple endocrine organs (2, 3).

Classical APS-1 typically presents in childhood or adolescence, and the clinical triad includes chronic mucocutaneous candidiasis (CMC), hypoparathyroidism, and primary adrenal insufficiency (PAI; Addison's disease). The clinical spectrum is broad, and an adjunct diagnostic triad of intermittent rash with fever (called APS-1 rash), autoimmune enteropathy, and enamel hypoplasia may aid earlier diagnosis (4, 5). Other manifestations including early-onset primary ovarian insufficiency (POI), vitiligo, and alopecia are also common (2, 4). Diagnosis of classical APS-1 is based on clinical findings, genetic testing for *AIRE* mutations, and screening for autoantibodies against specific cytokines and key proteins (often enzymes) in the targeted endocrine organs like 21-hydroxylase (21-OH) in the adrenal cortex and NACHT leucine-rich-repeat protein 5 (NALP5) in the parathyroid glands (6, 7). Autoantibodies against type I interferons (IFN)-Ia, in particular IFN-α and IFN-ω, are highly sensitive diagnostic markers present in almost all patients (8-11). Emerging evidence also highlights a role for elevated levels of IFN-γ and its downstream pathways in driving autoimmune tissue destruction (12, 13).

Classical APS-1 is rare, with a prevalence of <1:100 000 in most reports, although higher prevalence is found in genetic isolates, eg, 1:25 000 among Finns (NM_000383.4(AIRE):c.769C > T) and 1:9000 among Persian Jews (NM_000383.4(AIRE):c.254A > G) (14, 15). Over 150 disease-causing mutations in *AIRE* have been reported (16). While genotype-phenotypic correlations are limited, a low prevalence of CMC has been reported among Persian Jews (15), and we have previously described a splice site mutation with milder disease with later onset (17). Dominant negative *AIRE* mutations lead to nonclassical APS-1, which present with a mild and variable autoimmune phenotype resembling autoimmune polyendocrine syndrome type-2, often presenting with vitiligo and pernicious anemia (3, 18-21). Furthermore, coding variants in *AIRE* have been linked to increased risk of isolated adrenal insufficiency, type 1 diabetes, and pernicious anemia (22, 23). The wide range of onset, severity, and incomplete penetrance highlights the diverse clinical expression of *AIRE* mutations.

The Norwegian registry of organ-specific autoimmune diseases (ROAS) established in 1996, contains longitudinal follow-up data on classical APS-1 patients spanning nearly 30 years. Here we present a comprehensive analysis of clinical progression, *AIRE* genotypes, autoantibody profiles, cytokine signatures, and mortality in a national APS-1 cohort. Building on previous investigations (8, 24-30), we aim to better understand disease heterogeneity and inform long-term management strategies.

## Materials and methods

### Sex as a biological variable

Our study examined male and female humans, and sex was considered as a biological variable.

### Patients

Patients with classical APS-1 (n = 49) and nonclassical APS-1 (n = 22) enlisted in ROAS between 1996 and 2025 were included. The diagnosis of classical APS-1 was confirmed by the presence of biallelic *AIRE* mutations, while nonclassical APS-1 was defined by the presence of a single *AIRE* mutation with a proven dominant negative effect on AIRE function by an in vitro cell-based assay previously reported (3, 21). In short thymus-like 4D6 cells were cotransfected with different ratios of wildtype and mutated *AIRE* followed by assay of the transcription of AIRE-regulated genes. If the mutant was able to inhibit the transcription of AIRE-regulated genes by the wildtype transcript, the mutant was classified as dominant.

Data from patients with autoimmune PAI without APS-1 (n = 999) registered in ROAS were used to compare onset of PAI and mortality with APS-1 patients. All autoimmune PAI patients were negative for disease-causing mutations in *AIRE* evaluated either by Sanger or exome sequencing.

### Clinical data collection

Patient data was retrieved from ROAS, including clinical data reported by the physicians and specialists in accordance with national diagnostic guidelines, as well as clinical chemistry laboratory tests and findings of autoantibodies. Dental examinations were performed on 42 APS-1 patients with special attention to enamel hypoplasia by a specialist in cardiology.

Patient collection has been ongoing since 1996, hence spanning close to 30 years of longitudinal data for many of the patients. For the diagnoses POI and enamel failure, the registration spans fewer years. Regarding the nonclassical APS-1 patients, data were available from 2015 when the first patients were reported.

The age of onset of PAI was assessed in 999 patients without APS-1, diagnosed with either isolated PAI or autoimmune polyendocrine syndrome type 2 (APS-2), and in 47 APS-1 patients with confirmed PAI. Twelve APS-1 patients diagnosed with PAI but lacking a documented age at diagnosis were excluded. These data were then used to compare the age of onset of PAI between patients with and without APS-1.

### Assay of autoantibodies

Autoantibodies against 21-OH, 17-OH, glutamic acid decarboxylase, side-chain cleavage enzyme, tryptophan hydroxylase, tyrosine hydroxylase, aromatic L-amino acid decarboxylase, IFN-ω, NALP5, interleukin (IL)-17F, and IL-22 were assayed by radioimmunoassay as previously described (10). Autoantibodies against insulin (Catalog# DE7430, RRID:AB_3717490) and intrinsic factor (Catalog# DE7140, RRID:AB_3717491) were measured by ELISA (all from Demeditec Diagnostics GmbH, Germany).

### Assay of cytokines

The concentrations of cytokines IFN-α (multiple isoforms), IFN-β, IFN-γ, IFN-λ (λ1 and λ2), IL-1α, IL-4, IL-5, IL-6, IL-8, IL-10, IL-12, IL-13, IL-15, IL-17, IL-23, and TNF-α were assayed in serum from 34 APS-1 patients and 29 blood donors with a 16-plex VeriPlex Cytokine Multiplex ELISA test (PBL Assay Science, Catalog# 51510-1, RRID:AB_3717493) according to the manufacturer's instructions. The plate was imaged with a Chemidoc XRS (Bio-Rad) and quantified using Q-View v.207 software (Quansys Biosciences).

A Luminex assay was employed to validate the most interesting findings of the Veriplex assay and to explore additional targets recently highlighted by Oikonomou et al (12). The concentration of cytokines [IFN-α, IFN-γ, IL-1α, IL-6, IL-8, IL-10, IL-23, IL-17A, CD137, C-X-C motif chemokine ligands (CXCL)9, and CXCL10] were measured in serum from 47 patients with classical APS-1, 22 with nonclassical, and 49 blood donors with a custom Human Luminex Discovery Assay (Bio-Techne Ltd, Catalog# LXSAHM, RRID:AB_2924693, assay ID: x8mjhcvH) according to the manufacturer's instructions. The fluorescent intensity data were read on a Luminex 200 (DiaSorin SpA) instrument. The blood donor cohort is different for the Luminex and the Veriplex assays, while there is a large overlap for the classical APS-1 patients.

To assess the effects of prolonged disease duration on immune activation in APS-1 patients, we measured soluble receptor isoform of IFN-β (sIFNAR2; PBL Assay Science, Catalog# 41385, RRID:AB_3717494) and neopterin (IBL International, Catalog# RE59321, RRID:AB_3717495) in serum from 37 APS-1 patients and 29 blood donors with ELISA according to the manufacturer's instructions.

### Statistics

Analyses of description and comparison of demographic characteristics, clinical manifestations, and mutational profiles in APS-1 were performed in R (version 4.3.0). For the cytokines, sIFNAR2 and neopterin measurements, nonparametric statistical tests were employed due to the non-Gaussian distribution of the data. For Luminex, group differences were assessed using the Kruskal-Wallis test, followed by Dunn's multiple comparisons test, to identify pairwise differences between the 3 groups. A Mann-Whitney U test was used for the VeriPlex assay. All aforementioned tests were performed in GraphPad Prism 10.4.1. To account for multiple testing across the cytokine panels, false discovery rate correction was applied to all *P*-values using the Benjamini-Hochberg procedure implemented via the p.adjust function in R (version 4.5.1), and statistical significance was defined as false discovery rate-adjusted *P* < .05.

### Study approval

All participants signed informed consent prior to participation, and the study was approved by the Regional Committee for Medical and Health Research Ethics (approval numbers 2009/2555 and 2013/1504). Healthy controls used for autoantibody and cytokine assays were recruited anonymously upon informed consent through the blood donation unit at Haukeland University Hospital.

## Results

### The Norwegian APS-1 patient cohort

Altogether, 71 APS-1 patients (33 females) from 44 families with either classical (n = 49, 20 females, 38 families) or nonclassical APS-1 (n = 22, 13 females, 6 families) were included. All patients with classical APS-1 had the classical dyad (at least 2 out of the 3 cardinal manifestations). Classical APS-1 had an earlier debut age (12 ± 12 vs 20 ± 17) and a higher average number of clinical manifestations (5 ± 2 vs 2 ± 2) (Table 1) compared with nonclassical APS-1. Overall, 32 patients (45%) had at least 1 family member diagnosed with APS-1, more frequent among classical (24 of 49 patients, 49%) compared with nonclassical APS-1 (8 out of 22 patients, 36%).

**Table 1:** Demographics of Norwegian APS-1 patients

	Classical APS-1	Nonclassical APS-1	All APS-1
Number of patients	49	22	71
Sex (female/male)	20, 29	13, 9	33, 38
Number of families	38	6	44
Clinical debut age (yr) (mean ± SD)	12 ± 12	20 ± 18	13 ± 14
Number of deceased	15	2	17
Age at death (mean ± SD)	43 ± 19	80 ± 20	84 ± 16
Manifestations (median, range)	5 (1-11)	1 (0-7)	5 (0-11)

Abbreviation: APS-1, autoimmune polyendocrine syndrome type 1.

### Clinical manifestations

**Figure 1:**
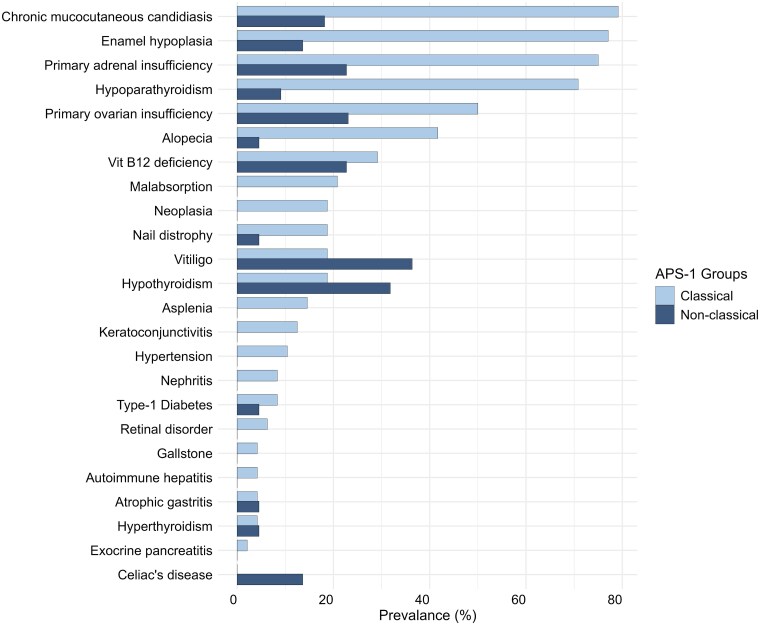
Prevalence of disease components in classical and nonclassical APS-1. Manifestations of classical (upper bar, n = 48 patients) and the nonclassical APS-1 (lower bar, n = 22 patients) ordered by prevalence in classical APS-1. Abbreviation: APS-1, autoimmune polyendocrine syndrome type 1.

Among the classical APS-1 patients (n = 48), the most common clinical manifestations were CMC (79%) and enamel hypoplasia (77%), followed by PAI (75%) and hypoparathyroidism (71%), making enamel hypoplasia the most common nontriad manifestation (Fig. 1). Overall, the complete clinical triad was observed in 44% of the patients (Table 2). Other common manifestations were POI, alopecia, vitiligo, hypothyroidism, and vitamin B12 deficiency (Fig. 1, Table 2). We also observed more uncommon manifestations such as hyperthyroidism (Grave's disease), exocrine pancreatic insufficiency, autoimmune hepatitis, and optic neuritis. The earliest clinical presentation was within the first year of life, observed for hypoparathyroidism and CMC (Table 2). The 3 components of the diagnostic triad, along with nail pitting, had the lowest average ages of onset (below 20 years for all).

**Table 2:** Prevalence, age of onset, and sex distribution of disease components in Norwegian APS-1 cohort

Disease components*^a^*	Classical APS-1 (n = 48)							Nonclassical APS-1 (n = 22)						
	Prevalence*^b^*		Age at onset			Sex		Prevalence		Age at onset			Sex	
	n	%	Mean (±SD)	min	max	M % (n)	F % (n)	n	%	Mean (±SD)	min	max	M % (n)	F % (n)
Classical triad														
Primary adrenal insufficiency	36	75	15.2 ± 9.4	4	55	79 (23)	65 (13)	5	23	21.2 ± 5.7	12	26	22 (2)	23 (3)
Chronic mucocutaneous candidiasis	38	79	17.7 ± 16.9	0	64	79 (23)	75 (15)	4	18	—	—	—	22 (2)	15 (2)
Hypoparathyroidism	34	71	13 ± 12.2	0	60	69 (20)	70 (14)	2	9	6 ± 1	5	7	0	15 (2)
Complete triad	21	44				48 (14)	35 (7)	0	0	—	—	—	0	0
Other endocrine diseases														
Hypothyroidism	9	18	37.3 ± 16.2	16	55	10 (3)	30 (6)	7	32	41.8 ± 17.8	21	70	22 (2)	38 (5)
Primary ovarian insufficiency*^c^*	10	50	22 ± 8.7	13	44	0	50 (10)	3	23	27.3 ± 10.5	15	40	0	23 (3)
Diabetes mellitus type 1	4	8	32.8 ± 15.3	13	54	7 (2)	10 (2)	1	5	34 ± 0	34	34	11 (1)	0
Hyperthyroidism	2	4	26 ± 11	15	37	0	10 (2)	1	5	36 ± 0	36	36	0	8 (1)
Gastrointestinal disorders														
Vitamin B12 deficiency/pernicious anemia	14	29	38.8 ± 20.4	13	74	34 (10)	20 (4)	5	23	31 ± 4	27	35	11 (1)	31 (4)
Malabsorption	10	21	18 ± 5.7	10	23	17 (5)	25 (5)	0	0	—	—	—	0	0
Autoimmune atrophic gastritis	2	4	16 ± 0	16	16	7 (2)	0	1	5	39 ± 0	39	39	0	8 (1)
Celiac's disease	0	0	—	—	—	0	0	3	14	42 ± 1	41	43	0	23 (3)
Autoimmune hepatitis	3	6	21 ± 20.8	2	50	7 (2)	5 (1)	0	0	—	—	—	0	0
Exocrine pancreatic insufficiency	2	4	35 ± 10	25	45	3 (1)	5 (1)	0	0	—	—	—	0	0
Skin diseases														
Alopecia	20	42	19.2 ± 9.9	4	40	48 (14)	30 (6)	1	5	7 ± 0	7	7	0	8 (1)
Alopecia totalis	6	13	17.4 ± 8.2	4	27	17 (5)	5 (1)	0	0	—	—	—	0	0
Vitiligo	10	21	23.3 ± 10.6	11	41	24 (7)	15 (3)	8	36	23.5 ± 17.2	8	60	22 (2)	46 (6)
Eye diseases														
Keratoconjunctivitis	6	13	17.6 ± 9.9	3	28	14 (4)	10 (2)	0	0	—	—	—	0	0
Optic neuritis	4	8	24 ± 2	22	26	3 (1)	15 (3)	0	0	—	—	—	0	0
Iridocyclitis	2	4	—	—	—	3 (1)	5 (1)	1	5	—	—	—	11 (1)	0
Other disorders														
Enamel hypoplasia	37	77	27.4 ± 19.4	6	68	66 (19)	90 (18)	3	14	27 ± 12	15	39	0	23 (3)
Nail pitting	9	19	12.4 ± 8.2	4	25	17 (5)	20 (4)	1	5	22 ± 0	22	22	0	8 (1)
Asplenism	7	15	34.6 ± 13.2	14	53	14 (4)	15 (3)	0	0	—	—	—	0	0
Nephritis	4	8	—	—	—	3 (1)	15 (3)	0	0	—	—	—	0	0

Abbreviations: APS-1, autoimmune polyendocrine syndrome type 1; F, female; M, male.

^*a*^Clinical manifestations were reported to the Norwegian Registry of Organ-specific Autoimmune Diseases by treating physicians at participating centers.

^*b*^Prevalences of classical APS-1 patients were calculated among cohort of 48 patients, as 1 individual was excluded due to passing away in the early childhood stage before the manifestations developed.

^*c*^Among females. Out of 20 females in classical APS-1, out of 13 females in nonclassical APS-1.

In the nonclassical APS-1 cohort (n = 22), vitiligo (36%) was the most common manifestation, followed by hypothyroidism (32%) (Fig. 1, Table 2). Within the diagnostic triad, PAI (23%) was the most frequent component, followed by CMC (18%) and hypoparathyroidism (9%). The mean age of onset of nonclassical APS-1 was higher than classical APS-1 for most manifestations (Table 2).

When comparing clinical features between classical and nonclassical APS-1, skin disorders showed the most pronounced difference. Alopecia was significantly more common in classical (42%) than nonclassical APS-1 (5%), in contrast to vitiligo (Table 2). Diagnostic triad components were more prevalent in classical APS-1, with hypoparathyroidism showing the greatest difference (8:1), followed by CMC (4:1) and PAI (3:1). Enamel hypoplasia and nail pitting were also more commonly observed in the classical APS-1 cohort. The sex distribution of PAI was similar between isolated PAI and nonclassical APS-1 (59% females each), while classical APS-1 overall displayed a reversed pattern with male predominance (58% males, 42% females).

### Natural course of classical APS-1

**Figure 2:**
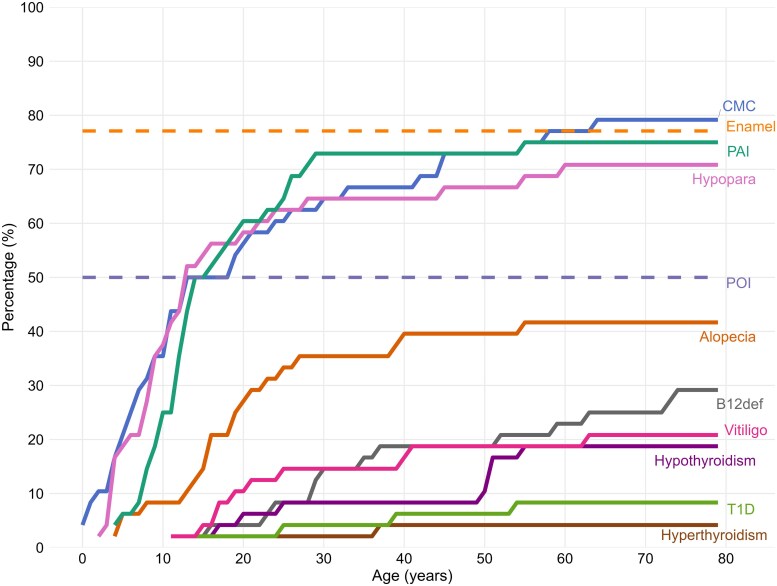
Age at diagnosis and life-long prevalence of APS-1 components in classical APS-1. Data from 48 patients with classical APS-1 are displayed. (For POI, prevalence among 20 female patients). Age at diagnosis was not available for POI and Enamel; hence they are represented as dotted lines indicating the prevalence in 2025 (POI: 50%, Enamel: 77%). Abbreviations: APS-1, Autoimmune polyendocrine syndrome type 1; B12def, vitamin B12 deficiency; Enamel, enamel hypoplasia; Hypopara, hypoparathyroidism; POI, primary ovarian insufficiency; T1D, type 1 diabetes.

**Figure 3:**
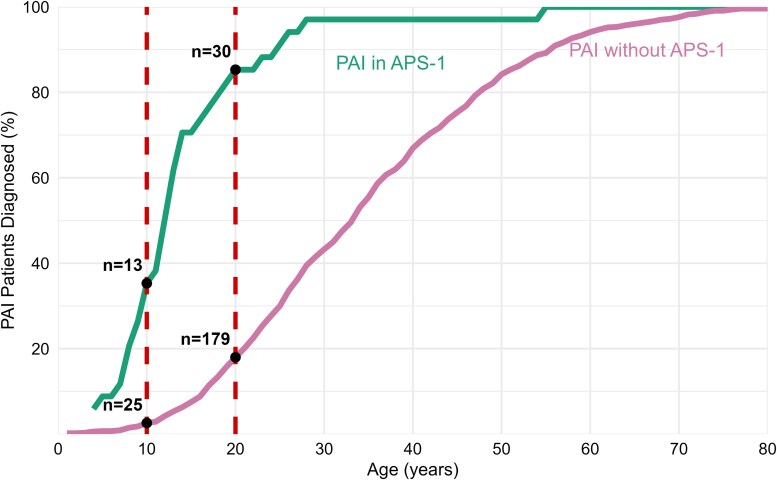
Ages of onset of autoimmune PAI with and without APS-1. In the APS-1 group, only patients with PAI were included (n = 35) and compared with autoimmune PAI without APS-1 (n = 999). Vertical lines show the number of patients diagnosed with PAI at 10 and 20 years of age, respectively. Abbreviations: APS-1, autoimmune polyendocrine syndrome type 1; PAI, primary adrenal insufficiency.

The Norwegian cohort of classical APS-1 patients has been followed for a period of up to 30 years. The 3 main components (CMC, PAI, and hypoparathyroidism) appear early in life, with the majority developing the diagnostic dyad by 20 years of age (n = 31, 63%). Furthermore, alopecia and vitiligo both had an early onset, reaching 27% and 10% of the cohort, respectively, by 20 years of age (Fig. 2). Although longitudinal data are scarce regarding enamel hypoplasia, it was present in 77% of the patients, underscoring the importance of awareness among dentists (Fig. 2). Looking at the age of diagnosis for PAI, 30 of 35 patients with classical APS-1 had received a PAI diagnosis by 20 years of age, whereas only 179 out of 999 patients with isolated PAI or APS-2 were diagnosed within the same age range (86% and 18%, respectively) (Fig. 3). APS-1 was overrepresented in early-onset autoimmune PAI, affecting one-third of patients diagnosed by 10 years of age (13/38) and one-seventh of those diagnosed by 20 years of age (30/209).

### Cancer and mortality

Neoplasms were diagnosed in 10 individuals (7 males and 3 females), all of whom had classical APS-1. Four patients had squamous cancer located in the oral cavity or lips, and 1 died of cancer-related complications. Two of these 4 patients were also diagnosed with neoplasms in the pelvic cavity (cervix and rectum). Oesophageal cancer was detected in 3 patients, all of whom are deceased (mean age at death 48 years ± 17 years). One patient was diagnosed with multiple myeloma and received curative treatment. The remaining patient had prostate cancer. Apart from the 9 patients with cancer, 1 patient had a benign papillary tumor located in the epiglottis.

**Figure 4:**
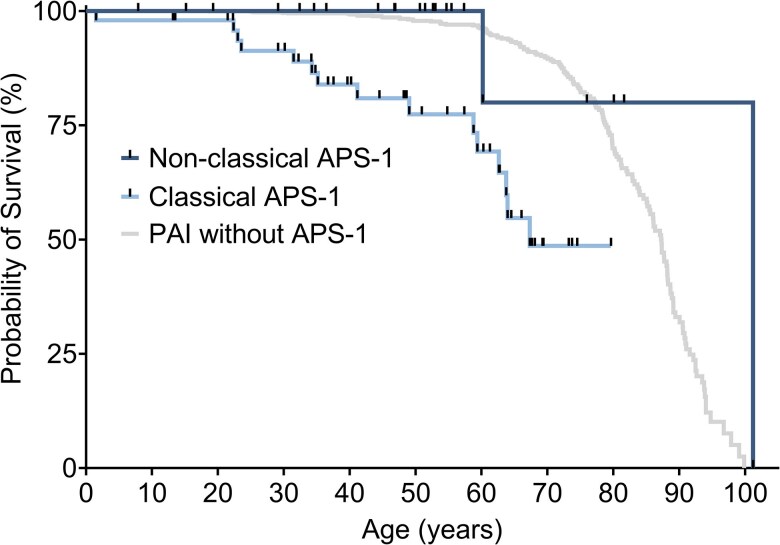
Survival curves. Kaplan-Meier survival analysis comparing patients with classical APS-1 (n = 49), nonclassical APS-1 (n = 22), and autoimmune PAI without APS-1 (n = 999). Follow-up extended from birth to January 1, 2025, or date of death. Vertical tick marks indicate censoring events (current age for living patients or administrative censoring at study end). Abbreviations: APS-1, autoimmune polyendocrine syndrome type 1; PAI, primary adrenal insufficiency.

Fifteen of 49 patients (31%) with classical APS-1 were deceased, with an average age of death of 42.7 years ± 19.3 (mean ± SD, median:41, range 2-68). Mortality appears to be higher among patients with classical APS-1 compared to dominant cases and autoimmune PAI without APS-1 (Fig. 4) and higher among males than females [Table S1, accession number S-BSST2658 (31)]. However, interpretation of the survival differences is complex as APS-1 is generally diagnosed earlier than PAI, which limits direct comparison of disease-specific mortality rates. The causes of death within the classical APS-1 group were cancer (27%), adrenal crisis (13%), cardiovascular pathologies (13%), sepsis-related mortalities (13%), renal failure (7%), diabetes with comorbidities (7%), dementia (7%), and unknown (7%) [Table S2, accession number S-BSST2658 (31)]. The nonclassical APS-1 cohort included 2 deceased individuals (1 male, 1 female, 9%), who died at 60 and 100 years of age. Causes of death for these patients were perioperative cardiac complications and old age, respectively.

### Genetic distribution/*AIRE* mutations in Norway

**Figure 5:**
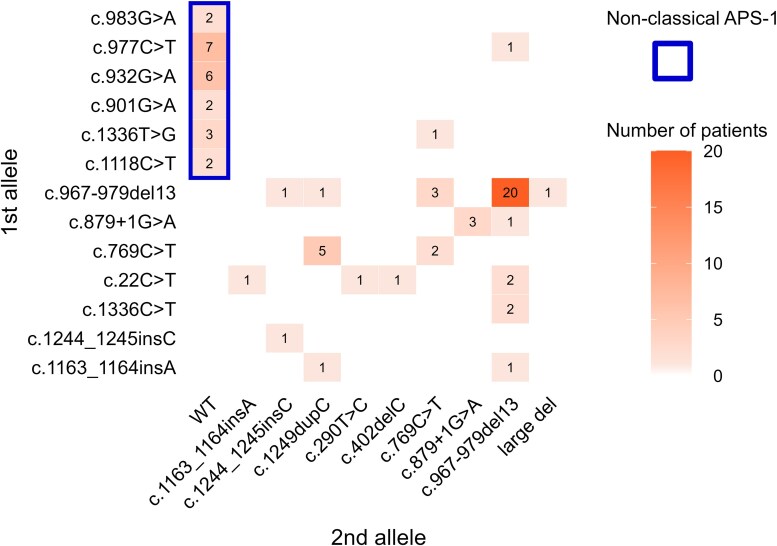
AIRE mutations in classical and nonclassical Norwegian APS-1 patients. Boxes marked with a frame indicate the nonclassical APS-1 patients carrying AIRE mutations with dominant-negative effect (n = 22 patients). Boxes without a frame show classical APS-1 patients (n = 49). All mutations refer to the MANE Select AIRE transcript NM_000383.4. Abbreviations: AIRE, autoimmune regulator; APS-1, autoimmune polyendocrine syndrome type 1.

All *AIRE* mutations and variants refer to the MANE Select transcript NM_000383.4. c.967-979del13 is the most frequently *AIRE* mutation in the Norwegian APS-1 cohort, with a total of 53 alleles (allele frequency 0.37) in 71 individuals. Screening 871 non-APS-1 PAI patients revealed 10 heterozygous carriers (allele frequency 0.0057), while 3 were identified within 360 healthy subjects (allele frequency 0.0042) (odds ratio = 1.38, *P* = .77). Other *AIRE* mutations in classical APS-1 included insertions (c.1163_1164insA, c.1244_1245insC, c.1249dupC), deletions (c.402delC, large del), single nucleotide variants (c.1336T > G, c.290T > C, c.769C > T, c.977C > T), and intronic variants (c.879 + 1G > A) (Fig. 5). All the dominant negative mutations were in the PHD1 domain of *AIRE*, except 1 (c.1336T > G) found in the PHD2 domain. Their dominant-negative effect in in vitro cell-based assays has been previously confirmed and reported (3, 21). As the predicted effects of the missense variants associated with nonclassical APS-1 and the proposed clinical classifications in databases such as ClinVar varies considerably [Table S3, accession number S-BSST2658 (31)], their deleterious function should always be confirmed by functional assays able to demonstrate dominant negative effects (3).

### Autoantibodies

**Figure 6:**
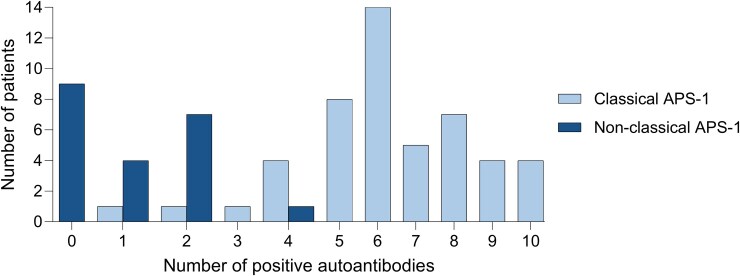
Distribution of autoantibody positivity among classical and nonclassical APS-1. Number of antibodies in patients with classical and nonclassical APS-1 based on assay of 12 different autoantibodies, including 21-hydroxylase, interferon-ω, side chain cleavage enzyme, 17-hydroxylase, glutamic acid decarboxylase, aromatic L-amino acid decarboxylase, tryptophan hydroxylase, tyrosine hydroxylase, NACHT leucine-rich-repeat protein 5, IL-17F, IL-22, and perilipin. One classical APS-1 patient was excluded due to death at 18 months of age without developing clinical or laboratory signs. Abbreviation: APS-1, autoimmune polyendocrine syndrome type 1.

Autoantibodies against IFN-ω were the most common serological marker in both patient groups (98% in classical vs 19% in nonclassical APS-1) (Table 3). The single patient without autoantibodies against IFN-ω was only 18 months at the time of testing. Autoantibodies targeting 21-OH, indicating autoimmune PAI, were the second most prevalent autoantibody, with 36 of 49 patients being positive in classical and 4 of 21 in nonclassical APS-1 (Table 3). Notably, 1 nonclassical APS-1 patient had PAI without detectable anti-21OH antibodies, and none of the patients in this group had autoantibodies against IL-17. Parietal cell autoantibodies were absent in the classical APS-1 group (Table 3). Most of the patients were positive for 4 or more autoantibodies (67% of all patients, 94% of classical APS-1), while 9 of the nonclassical patients had no autoantibodies (Fig. 6).

**Table 3.** Prevalence of organ-specific and cytokine autoantibodies associated with APS-1

Autoantibody against	APSI-1 type	Ab prevalence*^a^* (%)	Associated manifestation*^b^*	Ab-positive with manifestation (%)	Ab-positive without manifestation (%)
Interferon-ω	Classical	48/49 (98)	APS-1*^c^*	—	—
	Nonclassical	4/21 (19)		—	—
21-hydroxylase	Classical	36/48 (75)	PAI	32/35 (91)	4/13 (31)
	Nonclassical	4/21 (19)		4/5 (80)	0/16 (0)
17-hydroxylase	Classical	24/48 (50)	PAI	21/35 (60)	3/13 (23)
	Nonclassical	3/21 (14)		1/5 (20)	2/16 (13)
IL-22	Classical	38/48 (79)	CMC	34/38 (89)	4/10 (40)
	Nonclassical	1/21 (5)		0/4 (0)	1/17 (6)
IL-17F	Classical	14/48 (29)	CMC	12/38 (32)	2/10 (20)
	Nonclassical	0/21 (0)		0/4 (0)	0/17 (0)
NACHT leucine-rich-repeat protein 5	Classical	18/48 (38)	Hypoparathyroidism	16/33 (48)	2/15 (13)
	Nonclassical	1/21 (5)		2/2 (100)	0/19 (0)
Intrinsic factor	Classical	16/42 (38)	Pernicious anemia	6/12 (50)	10/30 (33)
	Nonclassical	3/18 (17)		3/5 (60)	0/13 (0)
Tryptophan hydroxylase	Classical	26/48 (54)	Enteropathy	6/9 (67)	20/39 (51)
	Nonclassical	3/21 (14)		0/0	3/21 (14)
Cholesterol side-chain cleavage enzyme	Classical	27/48 (56)	Ovarian insufficiency	8/10 (80)	19/38 (50)
	Nonclassical	1/21 (5)		1/3 (33)	0/18 (0)
Glutamic acid decarboxylase	Classical	24/48 (50)	Malabsorption	5/9 (56)	19/39 (49)
	Nonclassical	2/21 (10)		0/0	2/21 (10)
Insulin	Classical	1/42 (2)	Type 1 diabetes	0/4 (0)	1/38 (3)
	Nonclassical	2/18 (11)		1/1 (100)	1/17 (6)
Tyrosine hydroxylase	Classical	18/48 (38)	Alopecia	8/20 (40)	10/28 (36)
	Nonclassical	1/21 (5)		0/1 (0)	1/20 (5)
Aromatic L-amino acid decarboxylase	Classical	33/48 (69)	Autoimmune Hepatitis	3/3 (100)	30/45 (67)
	Nonclassical	2/21 (10)		0/0 (0)	2/21 (10)
Aromatic L-amino acid decarboxylase	Classical	33/48 (69)	Vitiligo	7/9 (78)	26/39 (67)
	Nonclassical	2/21 (10)		0/0	2/21 (10)
Perilipin	Classical	3/48 (6)	Lipodystrophy	NA	NA
	Nonclassical	3/21 (14)		NA	NA

Abbreviations: APS-1, autoimmune polyendocrine syndrome type 1; CMC, chronic mucocutaneous candidiasis; NA, not available; PAI, primary adrenal insufficiency.

^*a*^Prevalences of classical APS-1 patients were calculated among cohort of 48 patients, as 1 individual was excluded due to passing away in the early childhood stage before the manifestations developed. Not all patients have been tested for all autoantibodies.

^*b*^The most characteristic clinical associations reported in the literature. Individual autoantibodies may be present in patients with or without the corresponding manifestation.

^*c*^Interferon-ω is highly associated with APS-1; however, it is not specific to a single manifestation.

### Serum cytokine profiles of APS-1 patients

**Figure 7:**
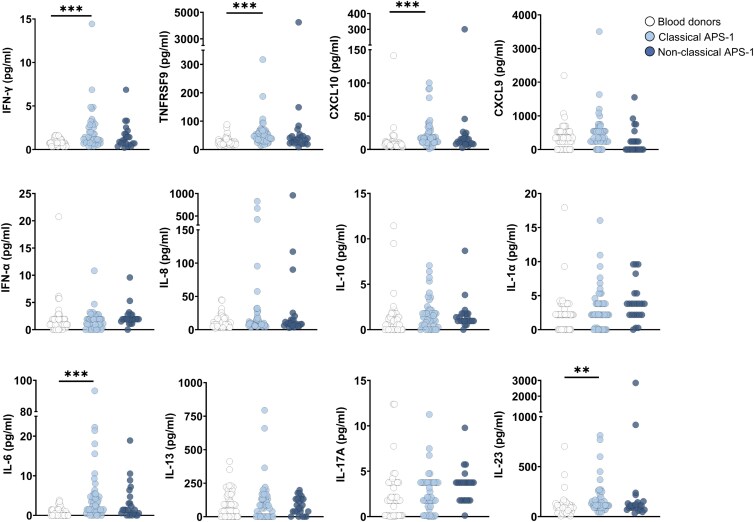
Serum cytokine profiling in APS-1 patients and healthy controls. Serum levels of cytokines were assayed with a custom Luminex assay in patients with APS-1 (classical: n = 47 and nonclassical: n = 22) and healthy blood donors (n = 49). Pairwise differences were tested with the Kruskal-Wallis test, followed by Dunn's multiple comparisons test. *P*-values were adjusted by an FDR test to account for multiple testing. *:*P* < .05, **:*P* < .01, ***:*P* < .001. Abbreviations: APS-1, autoimmune polyendocrine syndrome type 1; FDR, false discovery rate.

Serum levels of 12 pro- and anti-inflammatory cytokines were assayed. Cytokine selection was based on prior literature (12) and preliminary data comparing classical APS-1 patients with healthy blood donors [Fig. S1, accession number S-BSST2658 (31)]. We observed significantly elevated levels of the type II IFN-γ in classical APS-1 patients compared to both healthy blood donors and nonclassical APS-1 patients (Fig. 7). In contrast, IFN-α levels did not differ between groups. IFN-γ–inducible cytokines such as CXCL10 and TNFRSF9 (CD137) were also elevated in classical APS-1, whereas CXCL9 was not significantly elevated after adjusting for multiple testing.

The proinflammatory cytokine IL-6 was significantly increased in classical APS-1 and in several individuals with nonclassical APS-1, while the anti-inflammatory cytokine IL-10 and the Th2-associated IL-13 had similar levels across all groups. Other proinflammatory markers, including IL-1α and IL-8, which were elevated in the Veriplex assay [Fig. S1, accession number S-BSST2658 (31)], showed elevated levels in some patients across both APS-1 subtypes, but these differences did not reach statistical significance. Within the Th17 pathway, which is known to be dysregulated in classical APS-1 (32), IL-23 was markedly elevated, while IL-17A levels were not (Fig. 7).

**Figure 8:**
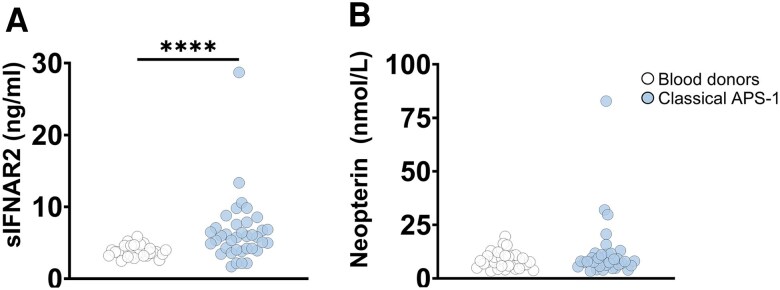
Serum cytokine profiling in APS-1 patients and healthy controls. Levels of sIFNAR2 (A) and neopterin (B) were assayed by ELISA in serum from patients with classical APS-1 (n = 37) and healthy blood donors (n = 29). Differences between groups assessed by a 2-tailed Mann-Whitney test. **** *P* < .0001. Abbreviations: APS-1, autoimmune polyendocrine syndrome type 1; sIFNAR2, soluble receptor isoform of IFN-β.

To further assess the downstream effects of the increased IFN signaling and the presence of IFN autoantibodies in classical APS-1 patients, we measured the soluble form of the IFN-α/β receptor, sIFNAR2. sIFNAR2 is known to be elevated in advanced cancer, hepatitis C, and multiple sclerosis patients receiving IFN-β treatment (33-36). Classical APS-1 patients had significantly higher levels of sIFNAR2 compared to healthy blood donors (Fig. 8A). Neopterin, a marker of monocyte/macrophage activation in response to IFN-γ and a biomarker of Th1-driven immune activation (37, 38), was clearly elevated in 3 APS-1 patients, but there was no difference at the group level (Fig. 8B). Notably, the patients with the highest neopterin level also exhibited the highest level of sIFNAR2, resulting in a moderate correlation between the 2 markers. However, when excluding this atypical patient from the analysis, the correlation was lost (Pearson's r² = 0.04, *P* = .82, n = 33), suggesting that the observed association was primarily driven by a single individual. Taken together, we found that patients with classical APS-1 had activation of inflammatory pathways, in particular IFN-γ and Th17.

## Discussion

This longitudinal study of 71 classical and nonclassical APS-1 patients reveals a huge variation in phenotypes, age of debut, disease course, and inflammation even among patients with the same *AIRE* genotype. These observations highlight the need to screen for APS-1 when organ-specific autoimmunity develops at a young age. Patients with nonclassical APS-1 exhibited a much milder clinical profile with greater interindividual variability and manifestations not aligning with the classical diagnostic criteria. These observations support the notion that a reduction in AIRE's functional capacity is associated with the degree of organ-specific autoimmunity (21).

In addition to the classical triad, enamel hypoplasia was a common clinical feature in our classical APS-1 cohort, consistent with previous reports diagnosing it in up to 70% of patients (4, 39, 40). Recent findings by Gropper and colleagues revealed that the enamel defect is associated with IgA autoantibodies targeting multiple enamel matrix proteins, leading to impaired development of permanent teeth (41). These findings indicate that enamel hypoplasia has an autoimmune cause and underscore the importance of regular dental follow-up for APS-1 patients. It also highlights the critical role of dentists in recognizing enamel hypoplasia as a potential early sign of APS-1.

Some patients with classical APS-1 are diagnosed late in life, often because they lack the typical triad of symptoms, present with only 1 major manifestation, or show only mild, nonendocrine autoimmune features. One example from our cohort is a family carrying a homozygous *AIRE* splice mutation showing late-onset, mild APS-1. One member developed PAI and CMC at 23 years, with enamel defects diagnosed later. Another was diagnosed with vitiligo at 15 years of age and type 1 diabetes after 30 years of age and later developed hypoparathyroidism and CMC. Both had protective human leukocyte antigen (HLA) types for PAI but tested positive for several APS-1-related autoantibodies, including IFN-ω, 21-OH, 17-OH, and NALP5 (17). Another patient developed his first manifestation (autoimmune PAI) at the age of 27, despite being homozygote for the common c.967-979del13 mutation in *AIRE*. APS-1 was only diagnosed because he was screened for IFN-ω autoantibodies. This highlights the challenges of diagnosing APS-1, and an adjunct triad has been proposed, focusing on “minor” components (APS-1 rash, enamel hypoplasia, and enteropathy) to facilitate early diagnosis (4, 5, 39, 42).

Patients with nonclassical *AIRE* variants generally present with a more complex, less severe, and less distinct phenotype more reminiscent of APS-2, making diagnosis more challenging (3, 21). Among the most frequent manifestations separating this cohort from classical APS-1 are vitiligo (36%), hypothyroidism (32%), and celiac disease (14%). Both hypothyroidism and celiac disease are often found at increased rates in patients with vitiligo (43, 44). We speculate that there is a strong genetic predisposition, especially since this cohort largely consists of a few families, strongly influenced by HLA haplotypes. A drawback of our data material is the lack of HLA data for this cohort. These variants are often identified through targeted *AIRE* sequencing prompted by clinical symptoms, inclusion in disease-specific biobanks or registries, or as candidate findings from clinical exome or genome sequencing. The functional relevance of the different heterozygous *AIRE* variants needs to be determined individually, and currently the best assessment is searching for a dominant negative effect of the variant using an overexpression system (3, 21). The dominant negative mutations are primarily located in the PHD-domains of *AIRE* where they disrupt protein oligomerization and impair function (3, 21). However, the potential effects of heterozygous mutations outside the PHD domains, particularly on AIRE's transcriptional activity and overall function, mostly lack experimental validation (18, 20, 45). In the Norwegian cohort, dominant negative mutations were detected in 2 families through disease registry screening for *AIRE* exon 8 variants, leading to their diagnosis with nonclassical APS-1. Hence, in patients presenting with autoimmune symptoms inconsistent with conventional diagnostic criteria, those with complex immunodeficiency profiles and families with accumulation of organs-specific autoimmune disease, screening for *AIRE* variants is recommended with nonclassical APS-1 as a potential underlying diagnosis.

Autoantibodies recognizing IFN-ω remain the most reliable screening marker for classical APS-1. Four individuals in our cohort were identified by screening for IFN autoantibodies and confirmed by finding disease causing mutations in *AIRE*. One such case involved a male diagnosed with isolated PAI at age 12, who tested positive for autoantibodies against 21-OH, side-chain cleavage enzyme (SSC), IFN-ω, and IL-22. At age 30, he still only had PAI but was found to carry a homozygous c.967-979del13 mutation in exon 8 of *AIRE* through a screening of early-onset PAI cases (46). His parents were confirmed heterozygous carriers. At 42, he developed CMC after recurrent candidiasis and an episode of esophageal obstruction. This case illustrates early-onset PAI with very late onset of other APS-1 features, particularly CMC, and that APS-1 should be suspected if PAI presents before the age of 20 years.

Cytokine profiling of classical APS-1 patients revealed a broad proinflammatory signature, with elevated levels of IFN-γ, IL-1α, IL-6, CXCL10, TNFRS9 (CD137), and IL-8 (CXCL8). While the 2 methods used to measure cytokines yielded some conflicting results, likely due to low serum concentrations or differences in assay sensitivity and isoform type (eg, IL-17A vs total levels of IL-17), the overall findings were consistent and complementary. The findings expand upon the IFN-γ–centric immune dysregulation previously described, identifying IFN-γ as a key driver of tissue inflammation in APS-1 (12). Elevated IL-23, IL-6, and IL-17 further point to the involvement of Th17-mediated responses, which we have previously shown to fail upon stimulation with *Candida albicans* (47). A dysfunction of the Th17 pathway has been suggested to explain the insufficient immune response toward *C. albicans* observed in patients with APS-1 (12, 47, 48) and are signs of chronic inflammation in APS-1.

Notably, we also observed increased levels of sIFNAR2, potentially reflecting an additional dysregulation of type I IFN signaling in addition to the autoantibodies. To our knowledge, this is the first report of elevated sIFNAR2 in autoimmune diseases. While previous studies have reported increased levels of sIFNAR2 in multiple sclerosis patients treated with IFN-β (49), these findings have largely been interpreted as a treatment-induced phenomena. In contrast, our data indicate that sIFNAR2 upregulation in classical APS-1 is intrinsic to the disease process itself, representing a disease-specific feature of IFN pathway dysregulation. This distinction highlights the potential of sIFNAR2 not only as a biomarker of interferon-induced inflammation but also as a functional modulator of interferon signaling. Supporting this, Novoa et al reported elevated sIFNAR2, but not sIFNAR1, in patients with severe COVID-19, further implicating sIFNAR2 as a dynamic regulator of IFN responses in systemic inflammatory states together with the neutralizing autoantibodies against type I IFNs (50). This further adds to the notion of dysfunctional IFN responses in APS-1 patients, where a downregulation of interferon-regulated genes has been observed (51, 52).

In conclusion, serum cytokine profiling reveals a broad proinflammatory signature, which highlights aberrant interferon levels associated with the classical type of APS-1. Our longitudinal follow-up of classical APS-1 reveals substantial variations in phenotype and disease course, highlighting that other factors, including genes and environmental factors, play important regulatory roles reflected by mild cases initially thought to be isolated autoimmune PAI or APS-2. Early debut of PAI is a strong indicator of classical APS-1, and therefore we suggest screening for APS-1 in cases of autoimmune PAI diagnosed before 20 years of age.

## Data Availability

Some or all datasets generated during and/or analyzed during the current study are not publicly available but are available from the corresponding author on reasonable request.
